# Multicentric validation of the proof of concept for utility of Trueprep extracted DNA in line probe assay testing

**DOI:** 10.1371/journal.pgph.0004422

**Published:** 2025-04-15

**Authors:** Priya Rajendran, Kumar Nishant, Vashistha Himanshu, Shanmugam Sivakumar, Kannan Thiruvengadam, Thirumalani Ramamoorthy, Reddy Kishore, Giri Sidhartha, Patel Pranav, Hanif Mahmud, Anand Sridhar, Ramachandran Ranjani, Kayesth Jyoti, Moore Moe, Hobson Reeti, Saini Sanjeev, Vadera Bhavin, Alavadi Umesh, Chandrasekaran Padmapriyadarsini

**Affiliations:** 1 ICMR - National Institute for Research in Tuberculosis (NIRT), Chennai, Tamil Nadu, India; 2 Central TB Division (CTD), New Delhi, India; 3 Infectious Disease Detection and Surveillance (IDDS) Project, ICF, New Delhi, India; 4 Regional Medical Research Centre (RMRC), Bhubaneswar, Odisha, India; 5 Intermediate Reference Laboratory (IRL), Ahmedabad, Gujarat, India; 6 New Delhi Tuberculosis Centre (NDTBC), New Delhi, India; 7 World Health Organization (WHO), New Delhi, India; 8 Infectious Disease Detection and Surveillance (IDDS) Project, ICF Incorporated LLC, Rockville, Maryland, United States of America; 9 United States of America Agency for International Development, New Delhi, India; PLOS: Public Library of Science, UNITED STATES OF AMERICA

## Abstract

In India’s National Tuberculosis Elimination Program (NTEP), diagnosing tuberculosis (TB) and Drug-Resistant TB (DR-TB) involves collecting two sputum specimens from presumptive TB patients. *Mycobacterium tuberculosis* (MTB) is detected using the Truenat MTB - RIF assay at peripheral sites, with Trueprep for DNA extraction. In the case of a MTB positive, the second specimen is sent to a linked reference laboratory for Line Probe Assay (LPA) testing, requiring DNA extraction again. This study aimed to validate Trueprep-extracted DNA for First Line (FL) and Second Line (SL) LPA testing to assess real-time programmatic field conditions and DNA transportation across diverse Indian geographies. Four hundred MTB-positive Trueprep extracted DNA with ≥10^4^ cfu/ml value from the first specimen, accompanied by the second sputum specimen, were transported from nine strategically selected Truenat sites (regions: South- 2, North- 2, East- 2, West- 3) to reference laboratories in a cool chain. The MTB-positive Trueprep-extracted DNA and the Genolyse-extracted DNA from the second specimen were subjected to FL and SL LPA testing, and the results were compared. Whole genome sequencing was performed for discordance resolution among mismatched Trueprep and Genolyse DNA results. Interpretable results were obtained in 397 (99.25%) of the 400 Trueprep-extracted DNA samples for FL LPA testing and 363 (90.75%) samples for SL-LPA testing. In comparison, Genolyse extracted DNA yielded 371 (99.46%) and 362 (97.05%) valid FL and SL LPA results, respectively, from 373 smear-positive specimens. Among 400 specimens, 160 were 10^5^, 122 of 10^6^, 101 of 10^4^, 15 of 10^7^, and 2 of 10^8^ cfu/ml value. High concordance (97.7% for FL, 99.5% for SL LPA) was observed between Trueprep and Genolyse DNA. This study validates Trueprep-extracted DNA for reliable FL and SL-LPA testing in field settings, potentially streamlining NTEP’s diagnostic process, reducing reference laboratory workload, eliminating duplicate DNA extractions, and reducing the turnaround time (TAT).

## Introduction

The National TB Elimination Programme (NTEP) envisages eliminating tuberculosis (TB) by 2025, five years before the deadline for achieving the Sustainable Development Goals [1]. An essential step in attaining this goal is the early diagnosis of TB. The NTEP addresses the substantial burden of TB through a comprehensive strategy by implementing an integrated diagnostic algorithm combining sputum smear microscopy, nucleic acid amplification test (NAAT) (Truenat MTB-RIF or GeneXpert MTB/RIF assays), radiological assessments (X-rays), Line Probe assay (LPA) and liquid culture and drug susceptibility testing (DST). With the expansion of NAAT sites (n=5090 in 2022), NTEP envisions replacing smear microscopy with upfront molecular testing using NAAT to diagnose TB at 8,000 high-workload microscopy facilities [2]. Strengthened diagnostic capacities at various levels of healthcare have improved early detection and treatment initiation by using this multi-faceted approach, demonstrating India’s commitment to tackling TB effectively.

In NTEP, upfront NAAT testing is recommended to offer Universal Drug susceptibility testing (UDST) to presumptive TB patients. Along with the GeneXpert assay, novel diagnostic tools like Truenat MTB-RIF assay help provide upfront UDST. Truenat assay is indigenously developed by Molbio Diagnostics Pvt. Ltd., Goa, India, as a molecular test kit for detecting TB and Rifampicin (RIF) resistance and recommended by the Indian Council of Medical Research (ICMR) [3]. As per the TB diagnostic algorithm (4), two sputum specimens are collected from presumptive TB patients for diagnostic work-up of TB and DR-TB patients. At peripheral labs, GeneXpert MTB/RIF assay and/or Truenat MTB-RIF testing detects *M. tuberculosis* (MTB) and Rifampicin (RIF) drug resistance in one sputum specimen, and the second specimen is transported to the linked reference laboratory for Line Probe Assay (LPA) testing [First Line (FL) LPA test involves identification of drug resistance to Rifampicin and Isoniazid drugs and Second Line (SL) LPA involves drug resistance testing for Fluoroquinolones and, second line injectables drugs (SLID)] for which the DNA is extracted again after processing of specimens requiring more time and resources (DNA extraction reagents/kits, and human resource).

Though the LPA results are expected to be reported within five days of specimen receipt [4], delays are observed in meeting this turnaround time (TAT) due to various inherent field level and operational challenges. To address these challenges, we proposed a stepwise approach to use the Trueprep DNA extracted at the field level for LPA testing in reference laboratories. In Phase I of the study conducted in 2022, we demonstrated the utility of using the Trueprep extracted DNA in LPA testing (establishment of proof of concept) at a reference laboratory [5]. Based on the satisfactory results, phase II (this study) was initiated at the multicentric level (reference laboratories from various parts of the country).

In the Phase I study, the main objective was to assess the utility of Trueprep DNA in LPA testing. Validation and impact assessment of using transported Trueprep extracted DNA for LPA First line and Second Line) in a programmatic field setting was not analyzed in Phase I. Hence, the main objectives of the Phase II study were:

1. To assess the feasibility of transporting DNA from decentralized Truenat testing sites to linked reference laboratories (i.e.,) for LPA testing.
2. To assess the impact of DNA transportation with a cool chain on the quality and quantity of DNA received and the LPA results at the LPA testing laboratory.
3. To assess the impact of using Truenat DNA for LPA in terms of reduction of TAT, cost, and work hours compared to the current TB diagnostic algorithm practices.

## Materials and methods

### Ethics statement

The study was conducted in accordance with ICMR’s Ethical guidelines of Biomedical research on Human Participants, 2017. Since the stored and decoded samples were used in this study and participants were not involved, approval for waiver of consent from the institutional ethics committee was obtained (NIRT IEC ID 2021-048).

This multicentric field validation study was conducted at four reference laboratories, namely the National Institute of Research in Tuberculosis (NIRT) Chennai, New Delhi Tuberculosis Center (NDTBC) Delhi, Regional Medical Research Center (RMRC) Bhubaneswar, and Intermediate Reference Laboratory (IRL) Ahmedabad representing the country’s geographies from South, North, East, and West regions. Specimens were received from nine Truenat sites linked to these reference laboratories. DNA extraction was done from the first specimen using the Trueprep method at the Truenat site, and the second sputum specimen was transported to the linked reference laboratory. **Orientation and training.** After the completion of Phase I, a two-day orientation session (from September 8, 2022, to September 9, 2022) was conducted at NIRT Chennai to orient the Laboratory Technicians (LTs) and Senior TB Laboratory Supervisors (STLS) of the participating reference laboratories and linked Truenat sites.

### Specimen/DNA transportation mechanism

Specimens were transported following the triple-layered packaging procedure, and the temperature was maintained at 2-8˚ C during transportation. Different mechanisms were followed at the sites.

#### IRL NDTB Centre, Delhi.

A human courier delivered the specimens on the same day once they were received at the Truenat sites [GTB hospital (15 km) and Jhandewalan chest clinic (5 km)].

#### IRL Ahmedabad.

The courier agency delivered the specimen from Truenat sites [DTC Dahod (200 km), DTC Desa (190 km), and DTC Aravalli (125 km)] to the reference laboratory on the second day of specimen receipt.

#### NRL RMRC Bhubaneswar.

District HQ Hospital (30 km) at Cuttack (10 km) collected and received all the specimens and stored them at 2-8˚ C until picked by LT from the reference laboratory once a week. The Truenat site in the Capital Hospital, Bhubaneswar, transported the Trueprep DNA to the reference laboratory as and when the second specimen was available. It was transported on the same day by a human courier.

#### NRL NIRT Chennai.

The specimen received at the Tiruvallur Truenat site (44km) was stored at 2-8 ˚C and couriered to the reference laboratory weekly. For the Pulianthope site (6 km), LT visited the site daily to collect the Trueprep DNA as and when the second specimen was available.

### Laboratory procedures

DNA extracted using the Trueprep method was subjected to FL and SL-LPA testing at the reference lab. The second aliquot of sputum specimen was subjected to FL-LPA and SL-LPA testing using the Genolyse Kit after the appropriate processing and DNA extraction method. Briefly, the sputum specimen was decontaminated by N- acetyl- L cysteine sodium hydroxide (NALC-NaOH) method [6] and subjected to fluorescence microscopy (FM) assay. DNA was extracted from the smear-positive specimens using the Genolyse reagent per the manufacturer’s LPA (FL and SL) testing protocol. Unlike the NTEP diagnostic algorithm, where only smear-negative specimens are subjected to culture, all specimens were subjected to liquid culture in this study. All smear-negative specimens were subjected to indirect LPA after the culture was positive (except for the Ahmedabad site, where DNA of only smear-positive specimens was used for LPA testing). The FL and SL-LPA results of the DNA extracted by Trueprep and Genolyse method were compared. The discordance between Truenat and LPA results obtained by either DNA extraction method was resolved through whole genome sequencing (WGS) using the Illumina platform. The WGS testing was outsourced to an external service provider (Genotypic Technology Pvt Ltd, Bengaluru).

### Statistical analysis

The data underwent verification for completeness and consistency and were analyzed using STATA software version 15.0 (Stata Corp., Texas, USA). The variables of interest were computed for frequency, percentage, median, minimum, and maximum. The time taken for specimen processing was analyzed to determine the significance of the distance to the specimen collection and processing center using the Kruskal - Walli’s test, followed by the Dunn test. The correlation between the quantity of DNA extracted by the Trueprep and Genolyse methods was assessed using Karl Pearson’s correlation coefficient. The difference was evaluated using the Mann-Whitney test, with and without the pairwise zero DNAs. A p-value of less than 0.05 was considered statistically significant.

## Results

**Fig 1:**
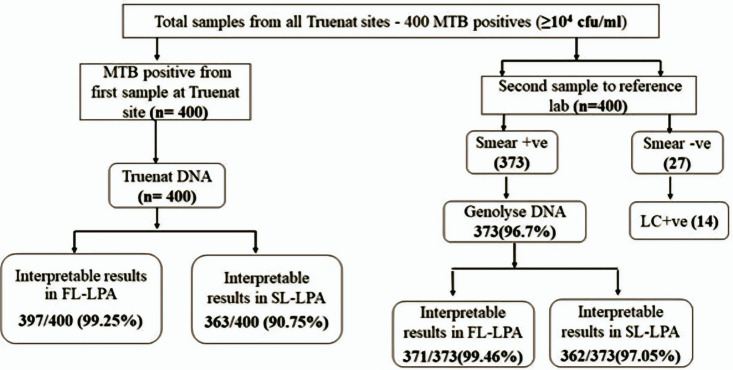
Consolidated LPA results for the Truenat MTB positive specimens with cfu/ml value ≥ 10^4^.

Four hundred Truenat MTB positive sputum specimens and their Truenat DNA received from the peripheral sites were used to assess the feasibility of using remnant Truenat DNA for LPA testing. Of these 400 processed specimens, 373 (93.25%) were smear-positive, and 27 (6.75%) were smear-negative. Of the 27 smear-negative specimens, 14(51.85%) were positive for MTB by liquid culture. Among 400 Truenat DNA, valid FL, and SL LPA results were available for 397 (99.25%) and 363 (90.75%). When the Genolyse results were explored among smear-positive specimens (n=373), 371 (99.46%) and 362 (97.05%) had valid FL and SL LPA results, respectively (Fig 1). Among smear-negative specimens, all 14 positive cultures had valid FL LPA and SL LPA results by Genolyse DNA testing.

### Site-wise analysis for resistance patterns

Only smear-positive specimens were included in the Ahmedabad site for FL and SL LPA testing. Among one hundred Genolyse and Truenat DNA, 87 were sensitive for INH and RIF, three were INH and RIF resistant, and one was RIF inferred and INH sensitive. There was a discordance in RIF inferred and INH resistance pattern accounting for three specimens in Truenat and two in Genolyse LPA testing. Six and five were found to be RIF sensitive and INH resistant by Genolyse and Truenat LPA testing, respectively. In SL-LPA testing, 86 and 84 were FQ and SLI sensitive using Truenat and Genolyse DNA, respectively, with one specimen being indeterminate and one specimen showing the resistance pattern of FQ inferred and SLI sensitive. Using both DNA, nine specimens were found to be FQ resistant and SLI sensitive (Table 1). In the Bhubaneswar site, among 100 (83 smear positive and 17 smear negative) Genolyse and Truenat DNA, 87 and 96 were RIF and INH sensitive, respectively. Four and five were RIF sensitive and INH resistant by Truenat and Genolyse testing. In SL LPA, 84 and 89 were found to be FQ and SLI sensitive using Truenat and Genolyse DNA. One specimen was FQ inferred SLI sensitive by both methods. One specimen was FQ and SLI resistant using Genolyse DNA and indeterminate by Truenat DNA. Seven specimens were found to be smear and culture negative. Among 98 smear-positive specimens, RIF and INH sensitive patterns were found in 90 Trueprep DNA and 88 Genolyse DNA in the Chennai site. RIF-sensitive INH-resistant pattern was found in 10 Trueprep DNA and 11 Genolyse DNA. One specimen was found to be smear-negative and culture-negative. In SL LPA testing, ninety-five were FQ and SLI sensitive by both methods. FQ inferred SLI sensitivity was seen in 3 specimens by both methods. One specimen was found to be FQ resistant and SLI sensitive by Genolyse, and two specimens were found to be indeterminate by Truenat (Table 1).

**Table 1:** Consolidated FL and SL LPA resistance patterns using Truenat and Genolyse.

			FL-LPA results From Trueprep									SL-LPA results from Trueprep							FL-LPA results From Genolyse								SL-LPA results from Genolyse					
**S.no**	**Reference Lab**	**Total Samples received**	**Valid results**	**RIF/** **INH (S/S)**	**RIF** **/INH (R/R)**	**RIF/** **INH (R/S)**	**RIF/** **INH** **(Inf/R)**	**RIF/** **INH (Inf/S)**	**RIF/** **INH (S/R)**	**MND**	**IND**	**Valid results**	**FQ/** **SLI (S/S)**	**FQ/** **SLI (R/S)**	**FQ/** **SLI (Inf/S)**	**MND**	**IND**	**Valid results**	**RIF/** **INH (S/S)**	**RIF/** **INH (R/R)**	**RIF/** **INH (R/S)**	**RIF/** **INH** **(Inf/R)**	**RIF/** **INH (Inf/S)**	**RIF/** **INH (S/R)**	**MND**	**IND**	**Valid results**	**FQ/** **SLI (S/S)**	**FQ/ SLI R/S**	**FQ/ SLI Inf/S**	**MND**	**IND**
**1**	**IRL NDTB Centre**	**100**	**98**	**85**	**5**	**2**	**0**	**0**	**6**	**0**	**2**	**85**	**84**	**1**	**0**	**0**	**15**	**95**	**81**	**4**	**3**	**0**	**0**	**7**	**0**	**0**	**92**	**89**	**3**	**0**	**0**	**3**
**2**	**IRL Ahmedabad**	**100**	**99**	**87**	**3**	**0**	**3**	**1**	**5**	**0**	**1**	**95**	**86**	**9**	**0**	**3**	**2**	**99**	**87**	**3**	**0**	**2**	**1**	**6**	**0**	**1**	**94**	**84**	**9**	**1**	**2**	**4**
**3**	**NRL RMRC Bhubaneswar**	**100**	**100**	**96**	**0**	**0**	**0**	**0**	**4**	**0**	**0**	**85**	**84**	**0**	**1**	**0**	**15**	**92**	**87**	**0**	**0**	**0**	**0**	**5**	**0**	**1**	**91**	**89**	**1** **(R/R)**	**1**	**0**	**2**
**4**	**NRL NIRT Chennai**	**100**	**100**	**90**	**0**	**0**	**0**	**0**	**10**	**0**	**0**	**98**	**95**	**0**	**3**	**0**	**2**	**99**	**88**	**0**	**0**	**0**	**0**	**11**	**0**	**0**	**99**	**95**	**1**	**3**	**0**	**0**
	**Total**	**400**	**397**	**358**	**08**	**02**	**03**	**01**	**25**	**0**	**03**	**363**	**348**	**11**	**04**	**03**	**34**	**385**	**343**	**07**	**03**	**02**	**01**	**29**	**0**	**02**	**376**	**357**	**14**	**05**	**02**	**09**

RIF – Rifampicin, INH – Isoniazid, S – Sensitive, R – Resistant, INF – Inferred, MND – MTB not detected, IND – Indeterminate

In the Delhi site, among 100 (92 smear positive and eight smear negative) Genolyse and Truenat DNA, 81 and 85 were RIF and INH sensitive, respectively. Four and five were resistant to RIF and INH by Genolyse and Truenat testing, respectively. The RIF-resistant, INH-sensitive pattern was found in two Trueprep and three Genolyse DNA. Six and seven were found to be RIF-sensitive INH resistant using Trueprep and Genolyse DNA. In SL LPA testing, 84 Trueprep and 89 Genolyse DNA were FQ and SLI sensitive. The FQ-resistant and SLI-sensitive patterns were found in two Trueprep and three Genolyse DNA, respectively (Table 1).

### Number of indeterminates

One indeterminate was reported from Ahmedabad, and two were from Delhi in FL LPA testing using Truenat DNA. In SL LPA testing, fifteen results were reported as indeterminate, each from Delhi and Bhubaneswar and two from Ahmedabad and Chennai, accounting for thirty-four indeterminates (8.5%). The number of indeterminates was lower when Genolyse DNA was used, accounting for only 2 (one from Ahmedabad and Bhubaneswar each) in FL LPA and 9 (2.2%) (2 in Bhubaneswar, three in Delhi, and 4 in Ahmedabad) in SL LPA testing.

### Discordance resolution

Although a high concordance (97.7% for FL, 99.5% for SL LPA) was observed between Trueprep and Genolyse DNA, nine specimens were found to have discordant results in FL LPA, with 1 in Chennai and Ahmedabad each, 3 in Delhi and 4 in Bhubaneswar (Table 2). Out of these nine specimens, eight cultures were available for DNA extraction and further sequencing. In SL LPA, two (1 from Ahmedabad and Delhi each) specimens were found to have discordance, and one specimen could not be sent for sequencing since the culture turned out to be negative.

**Table 2:** Sequencing results among the discordant sample.

S.no	Site	Specimen number	LPA testing	Resistance pattern	Sequenced result
1	NIRT Chennai	C01098	Genolyse DNA FL	RIF (S) INH (R)	INH (R)
			Trueprep DNA FL	RIF (S) INH (S)	
2	IRL NDTB Centre	D01175	Genolyse DNA FL	RIF (S) INH (R)	INH (R)
			Trueprep DNA FL	RIF (S) INH (S)	
3		D01121	Genolyse DNA SL	FQ (R) SLI (S)	FLQ (R)
				Trueprep DNA SL	FQ (S) SLI (S)
4,	IRL Ahmedabad	A02034	Genolyse DNA FL	RIF (S) INH (R)	RIF (S)
			Trueprep DNA FL	RIF Inf INH (R)	
5	NRL Bhubaneswar	B01072	Genolyse DNA FL	RIF (S) INH (R)	INH (R)
			Truenat DNA FL	RIF and INH (S)	
6		B01079	Genolyse DNA FL	RIF (S) INH (R)	INH (R)
				Truenat DNA FL	RIF and INH (S)
7		B02052	Genolyse DNA FL	RIF and INH (S)	INH (S)
				Truenat DNA FL	RIF (S) INH (R)
8		B02043	Genolyse DNA FL	RIF (S) INH (R)	INH (S)
				Truenat DNA FL	RIF and INH (S)

RIF – Rifampicin, INH – Isoniazid, S – Sensitive, R – Resistant, INF – Inferred

### Analysis based on the cfu/ml value

**Fig 2:**
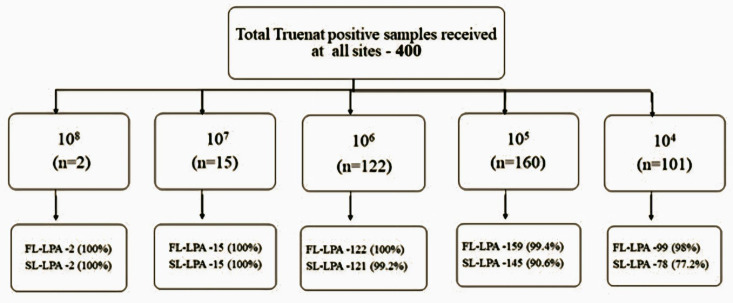
Break up of cfu/ml values for the specimens ≥ 10^4^.

**Fig 3:**
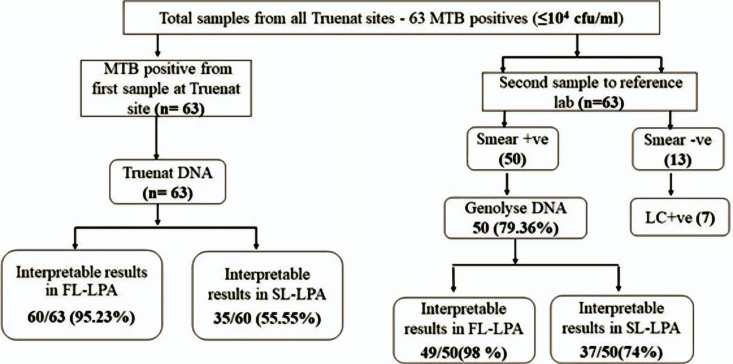
Consolidated LPA results for the Truenat MTB positive specimens with cfu/ml value < 10^4^.

**Fig 4:**
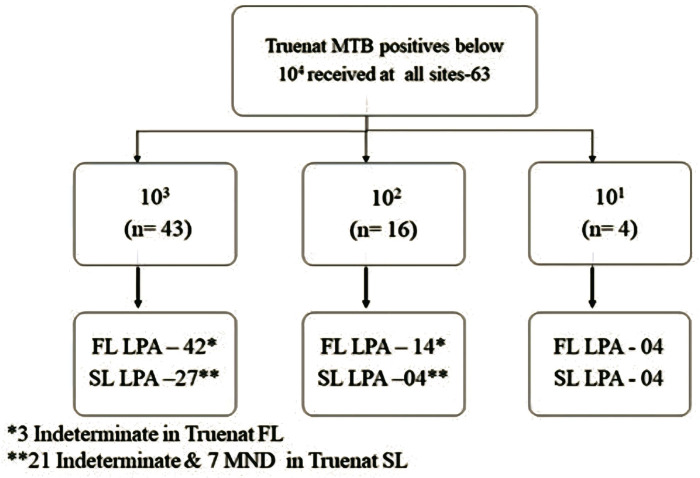
Break up of cfu/ml values for the specimens < 10^4^.

When the results of Trueprep DNA were analyzed based on the cfu/ml values, FL LPA had valid results for 98% of specimens with ≥10^4^ cfu/ml, whereas for SL LPA, valid results for more than 90% of the specimens were found with ≥10^5^ cfu/ml and 77.2% had valid results in DNA with 10^4^ cfu/ml (Fig 2). A subgroup analysis for Trueprep DNA with cfu/ml value <10^4^ (n=63) was conducted to assess the feasibility of using all MTB-positive DNA obtained from Truenat sites for LPA testing. Out of 63 Trueprep DNA evaluated, valid FL LPA results were found in sixty out of 63 (95.23%) compared to fifty-six out of 57 (98.24%) in Genolyse DNA. In SL LPA testing, 35 (55.5%) Trueprep DNA had valid results compared to 44 (77%) Genolyse DNA (Fig 3). Out of the 60 Trueprep DNA that gave valid FL LPA results, 42 were of 10^3^, 14 were of 10^2^, and 4 were of 10^1^ cfu/ml value. In SL LPA, out of 35 Trueprep DNA that gave valid results, 27 were of 10^3^, and 4 were of 10^2^ cfu/ml value (Fig 4).

### Turnaround time (TAT) analysis

#### Distance-wise analysis.

**Fig 5:**
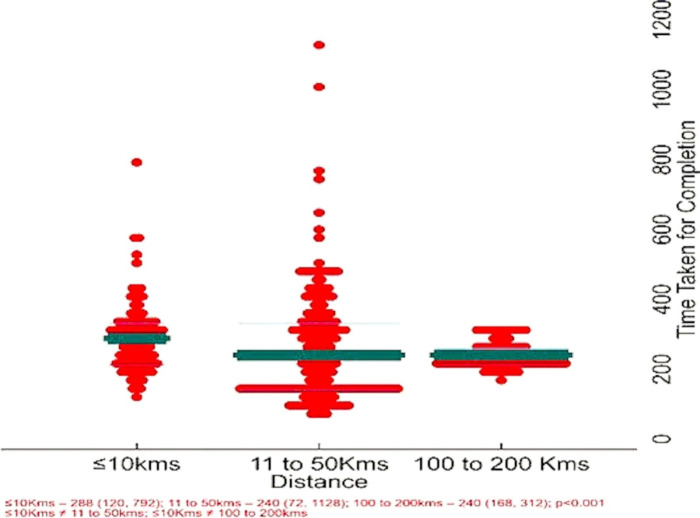
Distribution of the time taken for specimen processing, including specimen transport, from the Truenat site to the reference laboratory and report generation.

The time taken from sample receipt at the Truenat site to completing all procedures, including reporting, with the distance between the reference laboratory and the collection center was evaluated. The findings reveal that laboratories situated further away from the collection center, specifically over 100 kilometers, exhibited more efficient performance, with completion times ranging from 168 to 312 hours. Conversely, labs within a distance of less than 50 kilometers displayed a broader processing period, from 72 to 1128 hours. These disparities indicate statistical significance, confirming the influence of distance on the total time needed for the laboratory process. The average time, when calculated in days, was determined to be 12.1 (4.3) for distances of 10 km or less, 10.8 (6.6) for distances ranging from 11 to 50 km, and 10.1 (1.6) for distances of 100-200 km. The difference in TAT calculation was significant, with distances of 10 km or less having higher values than distances greater than 10 km (p<0.001) (Fig 5).

#### Report generation analysis.

The mean TAT from specimen reception at the reference laboratory to LPA reporting was 72 hours using Truenat DNA compared to 108 hours using Genolyse DNA in the Chennai site. Similarly, the difference was 81 and 126 hours using Truenat and Genolyse DNA at the Delhi site, respectively. At Gujarat and Bhubaneswar, the TAT was 105 and 124 hours using Truenat DNA and 156 and 179 hours using Genolyse DNA, respectively. The difference in TAT at these sites may be attributed to the laboratory’s existing workload for routine LPA testing, the availability of staff, functional equipment, and other operational issues in programmatic settings.

### Impact of transport on Trueprep DNA quantity

**Fig 6:**
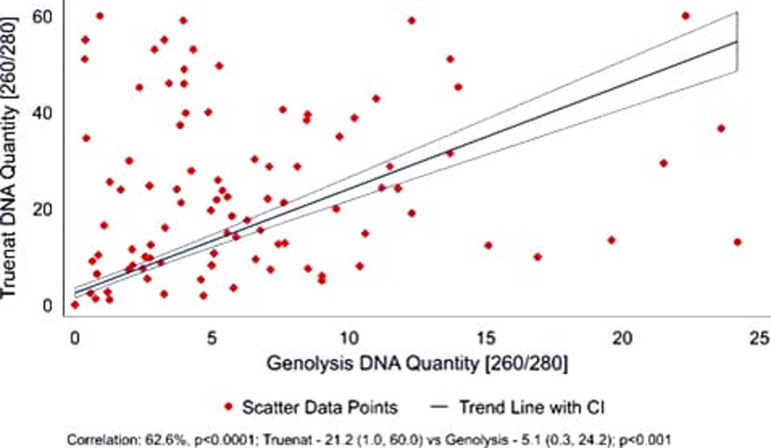
Comparison of the quantity of DNA extracted by Trueprep and Genolyse methods.

During transportation using a cool chain, the quality of the Trueprep DNA (elute)/sputum specimen did not compromise the results of the FL and SL LPA. The Quantity of Trueprep DNA obtained in Qubit data analysis ranged from 10-60 ng/µl. A pairwise correlation analysis was conducted to investigate the relationship between the quantity of DNA measured by Truenat and Genolyse. The data reveals a significant similarity of 62.6% (p<0.0001), indicating a substantial uniformity between the two methods in measuring DNA quantity. A Mann-Whitney test was performed to assess the difference in DNA quantity measured by Truenat and Genolyse. The median, minimum, and maximum observed DNA quantity obtained from Truenat and Genolyse were 21.20 (0.36, 24.20), and 5.12 (1.03, 60.00), respectively, after excluding pairwise zero DNA quantity. The results indicated a significant difference in DNA amount between the two methods, with Truenat DNA showing a statistically higher average quantity (Fig 6).

### Impact of using Trueprep DNA for LPA in terms of reduction in cost and work hours

**Fig 7:**
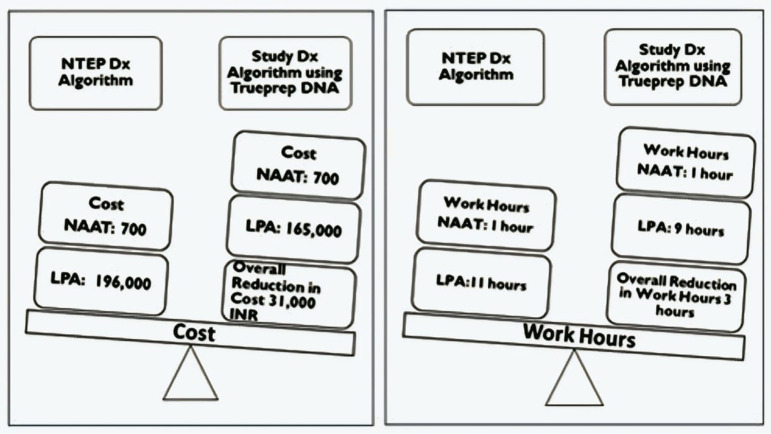
Reduction in cost and work hours due to Trueprep DNA utilization for LPA testing 1 USD ($) = 83 INR (as per 01 October 2023 conversion rate).

In routine diagnostic algorithms, the cost of specimen processing, DNA extraction, and LPA testing comes to 196,700 rupees (2,370$) for 100 specimens. When Trueprep DNA is used for LPA testing with no need for specimen processing, the cost gets reduced to 165,700 rupees (1,996$) with a difference of 31,000 (37.3$) between Truenat and Genolyse DNA testing for every one hundred specimens. Similarly, the work hours are also reduced to 10 hours when Trueprep DNA is used for LPA testing compared to 12 hours of LPA testing using Genolyse DNA, with a difference of 2 hours (Fig 7). In addition, under programmatic conditions, the time from sample receipt in the reference laboratory to processing and reporting of smear results takes 2-4 days, depending on the time the samples are delivered, the availability of staff, and functional microscopes. With Trueprep DNA being subjected directly to PCR, these 2-4 days can be saved.

## Discussion

Globally, research in TB diagnostics has gained attention through advocacy efforts, and there is an increased demand for rapid, quality-assured confirmatory diagnostic tools. In 2022, more than 3615 Truenat and 1200 GeneXpert machines were available in NTEP across the country to strengthen the decentralized TB testing using molecular diagnostics [2]. While DNA is not available at the end of the testing process in GeneXpert, about eighty-eight µl of DNA is accessible at the end of Truenat testing. As documented in ICMR literature on rapid health technology assessment in India [4], DNA isolated through a Trueprep device during Truenat assay is expected to be used for other WHO-recommended rapid diagnostic technologies (e.g., LPA) in resource-limited countries. Phase I of the study demonstrated proof of concept for the utility of Trueprep DNA in LPA testing under laboratory conditions, where we could obtain 97% of valid FL LPA results and 75% of valid SL LPA results using Trueprep DNA [5]. Based on the results and the number of indeterminates observed in Phase I, we determined a cutoff of ≥ 104 as the starting value for using Trueprep DNA for LPA testing under programmatic conditions. When the Trueprep DNA ≥ 10^4^ were subjected to LPA testing, 99.25% had valid FL LPA results compared to 99.46% from Genolyse DNA, demonstrating similar performance. However, there was a reduction in SL LPA interpretable results when tested from Trueprep DNA (90.75%) compared to Genolyse DNA (97.05%). Similar to the findings of Phase I [5] and a study from Lucknow [7], the number of indeterminates leading to a reduction of valid SL LPA results was higher when Truenat DNA was used (8.5%) compared to Genolyse DNA (2.2%). This could be attributed to several factors: the specimen processing method involved, the DNA extraction method, two different extractions from two other samples, personnel experimenting, and transport conditions of DNA from the Truenat site to the reference laboratory, given the fact that the bands in the SLLPA are lighter as compared to FLLPA and may be affected more by these factors. Moreover, in the Genolyse method, for smear-negative specimens, the source for DNA extraction is culture, which could have increased the number of valid results. Appropriate training and improvisation of processing methods (pretreatment of specimens with proteinase K) could reduce the number of indeterminates when Truenat DNA is used. In addition, the indeterminates, even after repeats, can be subjected to culture for further LPA testing. Genolyse DNA had higher concordance (7/8) with sequencing results than Trueprep DNA (1/8) when the discordant samples were subjected to sequencing. This could be attributed to the interpretation of the band patterns at different sites and warrants further research in the future.

When the number of specimens based on cfu/ml value was analyzed, FL LPA had valid results for nearly 98% of specimens with ≥10^4^ cfu/ml, whereas SL LPA valid results for more than 90% of specimens were found with ≥10^5^ cfu/ml and 77.2% had valid results in DNA with 10^4^ cfu/ml. As per the 2023 annual TB report, the number of Truenat tests performed countrywide in 2022 was 3,483,130, of which 5,29,196 (15%) were MTB positive. Assuming at least 75% (396,897 approx.) of the DNA of these MTB positives is with cfu/ml ≥ 10^4^, they can be sent for LPA testing with the advantage of evading the repeat of DNA extraction at reference labs. Similarly, among 27,519 (7,055 RR and 20,464 INH resistant) resistant specimens, 20,639 (75%) DNA with ≥ 10^4^ cfu/ml would be available for SL LPA testing. Moreover, since valid results were available for DNA with cfu/ml value <10^4^, the possibility of using them for LPA testing can be explored at least at a few specific sites. For smear-negative specimens, when subjected to culture, the time for LPA testing will range from 7 to 42 days. However, suppose the MTB-positive Trueprep DNA is used for LPA testing; the time for drug resistance diagnosis will get drastically reduced, resulting in earlier treatment of MDR and XDR patients.

The TAT, when analyzed based on the distance of specimen transport to the reference laboratory, revealed that faraway sites (100-200 km) had lower TAT compared to the nearer sites. This is because, at the Ahmedabad site, both specimens were obtained simultaneously and sent by a courier that reached the reference laboratory the next day. However, specific sites at Chennai and Bhubaneswar had to wait for the second specimen at least two days before it reached the reference laboratory. In addition, weekly transportation of specimens could also be the reason for increased TAT at these sites. However, when the TAT for report generation was analyzed, the Chennai site had the lowest TAT, followed by Delhi, Ahmedabad, and Bhubaneswar in LPA testing using Trueprep DNA compared to Genolyse DNA. The contrast in TAT for specimen transport and report generation could be attributed to the system adopted and the laboratory procedures followed at each site (weekly transportation of specimens for testing, availability of reagents and personnel, and holidays). The quantity of Trueprep DNA, when compared to Genolyse DNA, was higher in the former, indicating that the transport conditions did not have much impact. This shows that the Trueprep DNA, when transported under appropriate conditions, will not affect the LPA testing at the reference lab. Reducing work hours due to non-repeating specimen processing and DNA extraction is a significant advantage of using Trueprep DNA for LPA testing. In addition, the cost of reagents procured for specimen processing and DNA extraction can also be minimized. However, smear negatives, indeterminates, and DNA with cfu/ml value <10^4^ will need special attention and well-trained personnel. Incorporating a comprehensive approach to effective, accurate, and rapid TB diagnosis can help achieve early and effective treatment that will aid in filling the diagnostic gaps. For implementing outcomes of this study in programmatic settings, the utilization of Trueprep DNA for LPA testing in good-performing Truenat sites, at least in a few states, needs to be considered. Moreover, adequately monitoring the following parameters is essential to obtain reliable results.

a. Two specimens are being collected from a presumptive TB patient.
b. Trained personnel performing Truenat tests.
c. Efficient and quick transport of Truenat DNA to reference laboratory in cool chain
d. Timely delivery of DNA to the reference laboratory so that it can be subjected to further processing on the same day.
e. A systematic plan of LPA testing using Trueprep DNA within the stipulated time.

### Limitations of the study

a. Same-day or next-day delivery of the Trueprep DNA and second specimen was impossible at all sites. If properly planned, the DNA can be transported to the reference lab once it turns positive at the peripheral site, and the second specimen can be shifted later.
b. However, a significant limitation of the program will be that the DNA of MTB positives with cfu less than 10^3^, which would be about 25-30%, will not be subjected to LPA.
c. At some sites, the availability of Trueprep cartridges and MTB chips was hindered. Inventory management in coordination with CTD will help prevent such hiccups during the implementation of LPA testing using Trueprep DNA.
d. Cost savings were calculated only for LPA kits. However, substantial savings in working hours and electricity consumption for running the TB containment laboratory and equipment and its maintenance were not considered.

## Conclusion

Although we have demonstrated the feasibility of using Trueprep DNA for LPA testing in our earlier study, in this study, we have successfully executed the LPA testing using Trueprep DNA under programmatic conditions where the DNA from peripheral sites was transported to linked reference laboratories under appropriate temperature. We could also demonstrate the reduction of TAT, cost, and work hours compared to the current TB diagnostic algorithm practices. If the system is implemented in the program, patient management can be improved with earlier diagnosis of drug resistance.

## Supporting information

S1 Data Sequencing data for discordant samples. (XLSX)

S2 Data Statistics data for all sites. (XLSX)
